# Human DENND1A.V2 Drives *Cyp17a1* Expression and Androgen Production in Mouse Ovaries and Adrenals

**DOI:** 10.3390/ijms21072545

**Published:** 2020-04-06

**Authors:** Maria E. Teves, Bhavi P. Modi, Rewa Kulkarni, Angela X. Han, Jamaia S. Marks, Mark A. Subler, Jolene Windle, Jordan M. Newall, Jan M. McAllister, Jerome F. Strauss

**Affiliations:** 1 Department of Obstetrics and Gynecology, Virginia Commonwealth University, Richmond, VA 23298, USA; maria.teves@vcuhealth.org; 2 Department of Human and Molecular Genetics, Virginia Commonwealth University, Richmond, VA 23298, USA; bmodi@cmmt.ubc.ca (B.P.M.); kulkarnirm2@mymail.vcu.edu (R.K.); mark.subler@vcuhealth.org (M.A.S.); jolene.windle@vcuhealth.org (J.W.); 3 Department of Pathology, Penn State Hershey College of Medicine, Hershey, PA 17033, USA; Ah8703@pcom.edu (A.X.H.); jmarks5@pennstatehealth.psu.edu (J.S.M.); jnewell@pennstatehealth.psu.edu (J.M.N.)

**Keywords:** polycystic ovary syndrome, DENND1A, ovaries, adrenals, androgens, *Cyp17a1*

## Abstract

The *DENND1A* locus is associated with polycystic ovary syndrome (PCOS), a disorder characterized by androgen excess. Theca cells from ovaries of PCOS women have elevated levels of a *DENND1A* splice variant (DENND1A.V2). Forced expression of this variant in normal theca cells increases androgen biosynthesis and *CYP17A1* expression, whereas knockdown of the transcript in PCOS theca cells reduced androgen production and *CYP17A1* mRNA. We attempted to create a murine model of PCOS by expressing hDENND1A.V2 using standard transgenic approaches. There is no DENND1A.V2 protein equivalent in mice, and the murine *Dennd1a* gene is essential for viability since *Dennd1a* knockout mice are embryonically lethal, suggesting that *Dennd1a* is developmentally critical. Three different hDENND1A.V2 transgenic mice lines were created using CMV, *Lhcgr*, and TetOn promoters. The hDENND1A.V2 mice expressed hDENND1A.V2 transcripts. While hDENND1A.V2 protein was not detectable by Western blot analyses, appropriate hDENND1A.V2 immunohistochemical staining was observed. Corresponding *Cyp17a1* mRNA levels were elevated in ovaries and adrenals of CMV transgenic mice, as were plasma steroid production by theca interstitial cells isolated from transgenic ovaries. Even though the impact of robust hDENND1A.V2 expression could not be characterized, our findings are consistent with the notion that elevated hDENND1A.V2 has a role in the hyperandrogenemia of PCOS.

## 1. Introduction

Polycystic ovary syndrome (PCOS) is a common female endocrinopathy characterized by androgen excess. PCOS is the main cause of anovulatory infertility. PCOS thecal cells in culture show an increase in both the expression of the steroidogenic enzyme CYP17A1 and androgen secretion under basal conditions compared to theca cells from ovulatory women [1]. Multiple genes and factors have been implicated in the pathophysiology of PCOS [2]. The *DENND1A* locus has been associated with PCOS in diverse populations in genome-wide association (GWAS) and replication studies [3,4,5]. The *DENND1A* gene encodes a clathrin-binding protein that has an N-terminal guanine nucleotide exchange factor (GEF) function [6]. Clathrin is a major component of coated pits, where plasma membrane receptors cluster, including the receptors for gonadotropins and insulin, and are subsequently internalized and cycled through endocytic vesicles [7]. Thus, hDENND1A.V2 sits at the nexus of signaling of key hormones involved in reproduction [2].

An alternatively spliced transcript of the *DENND1A* gene, generating a truncated protein isoform that retains both the GEF and clathrin-binding domains, termed DENND1A.V2, is elevated in theca cells derived from women with PCOS [8]. Moreover, forced expression of hDENND1A.V2 in normal theca cells increases the expression of genes involved in androgen biosynthesis and androgen secretion, whereas knockdown of hDENND1A.V2 in PCOS theca cells reduced the expression of the steroidogenic genes and thus androgen production [8].

In cultured human theca cells, immunofluorescence studies revealed that DENND1A.V2 is co-localized with the LH receptor and the small GTPase, RAB5B, which is involved in vesicular trafficking. Additionally, hDENND1A.V2 and RAB5B were found to translocate into the nucleus, and nuclear accumulation is greater in cultured PCOS theca cells than in theca cells derived from ovaries of normal women [9]. This suggested that hDENND1A.V2 could act in the nucleus to control expression of steroidogenic genes and thus result in increased androgen biosynthesis. Collectively, these observations suggest that hDENND1A.V2 has a pathophysiological role in the hyperandrogenemia associated with PCOS.

There is considerable interest in establishing models of PCOS for examination of the basis of the reproductive as well as metabolic phenotypes that have been characterized in PCOS. The existing animal models of PCOS have been mainly produced by treating rodents with androgens [10,11] or inhibitors of aromatase, the enzyme that metabolizes androgens into estrogens [12]. PCOS-like phenotypes have also been produced by prenatally exposing sheep or rhesus macaque fetuses to androgens in utero [13,14]. To date, there have been no models established through manipulation of PCOS candidate genes identified through GWAS.

Since our previous studies on normal human theca cells established that elevating expression of DENND1A.V2 increased androgen production, we hypothesized that a hDENND1A.V2 transgene would augment endogenous androgen synthesis by mouse ovaries, creating a model of PCOS, or at least a phenocopy of the hyperandrogenemia and ovarian dysfunction of PCOS.

## 2. Results

### 2.1. hDENND1A.V2 Induces a PCOS Phenotype in Mouse Leydig MA-10 Cells—Evidence to Support a hDENND1A.V2 Mouse Model for PCOS

Based on the functional role of hDENND1A.V2 in human theca [8] and adrenal cells [15], we explored the potential of creating a transgenic mouse model expressing hDENND1A.V2. The mouse does not have a DENND1A.V2 equivalent transcript in public data bases. Therefore, we first examined the effects of forced expression of hDENND1A.V2 in mouse Leydig MA-10 cells to establish that hDENND1A.V2 could function in the context of a mouse steroidogenic cell [16,17]. *Cyp17a1* expression and steroid biosynthesis were measured in experiments on cells treated in the absence (C) or presence of 20 μM forskolin (F). As shown in Figure S1, hDENND1A.V2 adenoviral infection (3 pfu/10^6^ cells) of MA-10 cells significantly increased both basal and forskolin-stimulated *Cyp17a1* mRNA at 24 h, as compared to empty (Null) adenovirus. Seventy-two hours following adenoviral infection, forskolin-stimulated 17α-hydroxyprogesterone (17OHP4) and progesterone biosynthesis were significantly increased compared to the null virus controls, establishing that hDENND1A.V2 is functional in murine cells.

### 2.2. Generation of hDENND1A.V2 Transgenic Mice

**Figure 1:**
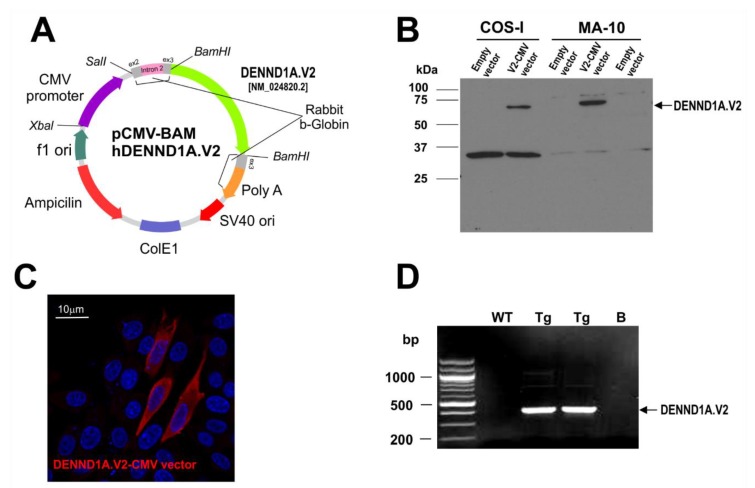
pCMV-BAM-hDENND1A.V2 expression construct. (**A**) Map for pCMV-BAM-hDENND1A.V2 construct. (**B**) Efficiency of the vector was tested by examining hDENND1A.V2 expression by Western blot and immunodetection in transfected COS-I and MA-10 cells. pCMV-BAM was used as the empty construct (negative control). (**C**) Immunofluorescence detection of hDENND1A.V2 in CHO cells transfected with the pCMV-BAM-hDENND1A.V2 construct. (**D**) Representative PCR results for the genotyping of wild-type and transgenic mice generated using the pCMV-BAM-hDENND1A.V2 vector. WT, wild-type; Tg, transgenic; B, blank; V2.CMV, pCMV-BAM-hDENND1A.V2 vector.

We attempted to create a PCOS animal model by expressing hDENND1A.V2 in mice using standard transgenic approaches. We first generated a pCMV-BAM hDENND1A.V2 construct using a CMV promoter to drive the expression of hDENND1A.V2 (Figure 1A). The efficiency of the vector was tested in transfected COS-I and MA-10 cells. As shown in Figure 1, cells were cultured and transfected with the pCMV-BAM hDENND1A.V2 construct and protein expression was subjected to Western blot and immunodetection using an anti-hDENND1A.V2 antibody [8,9] (Figure 1B). Expression of the protein was also detected in transfected CHO cell by immunofluorescence using the same antibody (Figure 1C). Transgenic animals were created as described in the -materials and methods section. Figure 1D shows the transmission of the transgene by PCR using genomic DNA. Transgene-positive mice show a single band of 421 bp.

**Figure 2:**
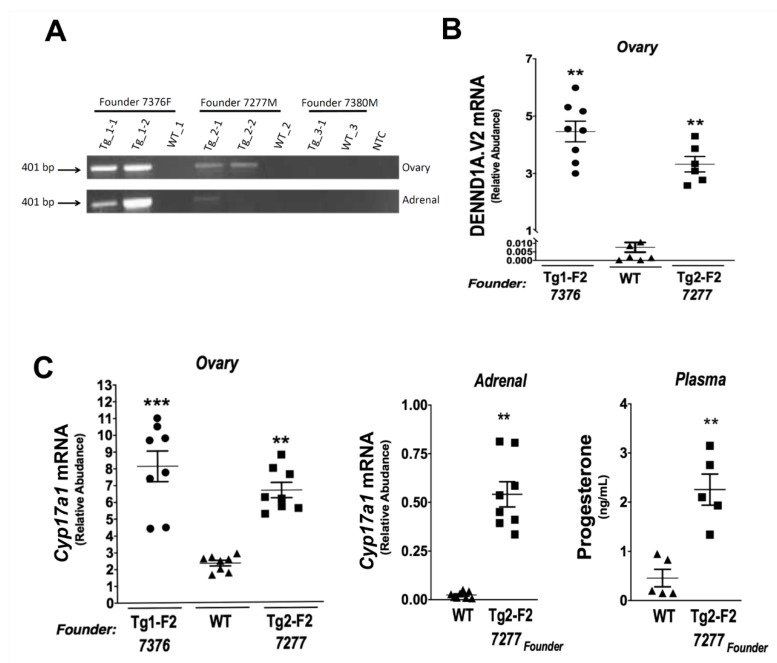
CMV-hDENND1A.V2 transgenic mouse profile. (**A**) Representative RT-PCR results from ovary and adrenal tissue collected from the three CMV-hDENND1A.V2 founder lines; 7376, 7277, and 7380. (**B**) Comparison of the relative expression of hDENND1A.V2 mRNA quantified by qRT-PCR in F2 generation ovaries collected from wild-type and CMV-hDENND1A.V2 7376 and 7277 founder transgenic mice, demonstrated elevated ovarian hDENND1A.V2 mRNA expression in Tg1-F2 7376 (**, *p*= 0.001) and Tg2-F2 7277 (**, *p*= 0.001), as compared to the wild-type mice. (**C**) In parallel studies, ovarian *Cyp17a1* mRNA levels were also elevated in Tg1-F2 7376 (***, *p* = 0.0001) and Tg2-F2 7277 (**, *p* = 0.0001) mice as compared to the wild-type mice (Figure 2C (left)). Adrenal Cyp17a1 mRNA was also increased in Tg2-F2 7277 mice (**, *p* < 0.001) as compared to the wild-type mice (Figure 2C (middle)). Progesterone levels were augmented in plasma from Tg2-F2 7277 mice (**, *p* < 0.001) (Figure 2C (right)). WT, wild-type; Tg, transgenic; F2, second generation.

Three founders were obtained using the pCMV-BAM hDENND1A.V2 construct (founder 7376F, 7277M, 7380M). Founders 7376F and 7277M progeny had hDENND1A.V2 transcript expression in the ovaries and adrenal tissue, detected by RT-PCR (Figure 2A) and qPCR (Figure 2B). These transgenic mice showed increased *Cyp17a1* mRNA levels in the ovary and adrenal compared to wild-type mice. Moreover, progesterone levels in plasma were higher in the pregnant mare’s serum gonadotropin (PMSG)/human chorionic gonadotropin (hCG)-treated hDENND1A.V2 transgenic mice compared to treated wild-type mice (Figure 2C). hDENND1A.V2 mRNA expression, quantified by qRT-PCR in F2 generation ovaries collected from the wild-type CMV-hDENND1A.V2 7376 (Tg1-F2 7276) and 7277 (Tg2-F2 7277) founder transgenic mice, demonstrated significantly elevated ovarian hDENND1A.V2 mRNA expression in seven Tg1-F2 7376 and six Tg2-F2 7277 mice compared to seven wild-type mice. Ovarian *Cyp17a1* mRNA levels were also significantly elevated in eight Tg1-F2 7376 and eight Tg2-F2 7277 mice as compared to seven wild-type mice (Figure 2C (left)). Moreover, adrenal *Cyp17a1* mRNA was also significantly increased in eight Tg2-F2 7277 mice, as compared to seven wild-type mice (Figure 2C (middle)). These results were unexpected since the *Cyp17a1* gene is not normally expressed in the mouse adrenals, which lack a functional adrenal reticularis zone [18]. As shown in Figure 2C (right), plasma progesterone levels were significantly augmented in CMV-hDENND1A.V2 7277 F2 mice, compared to wild-type mice.

**Figure 3:**
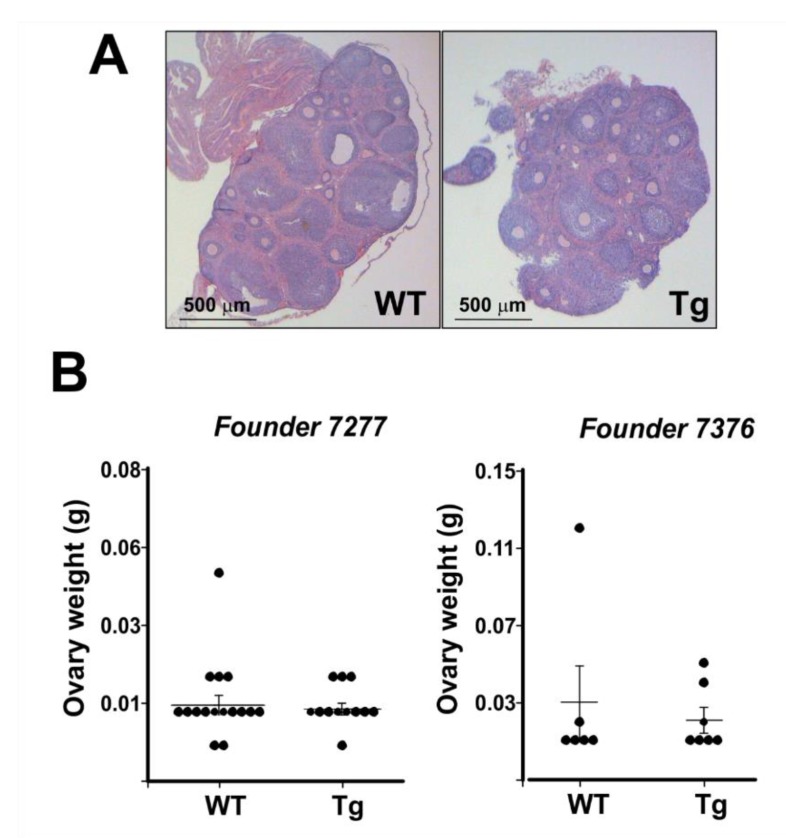
CMV-hDENND1A.V2 transgenic mice have morphologically normal ovaries. (**A**) Representative histological images from ovaries collected from wild-type and CMV-hDENND1A.V2 transgenic mice. (**B**) Measurements of the ovary weight from wild-type and transgenic mice for two of the founder lines. No significant differences were found in the histologic appearance and weights of PMSG/hCG-stimulated Tg and WT ovaries. For the 7277 founder, *p* = 0.73; for the 7376 founder; *p* = 0.61. WT, wild-type; Tg, transgenic.

**Figure 4:**
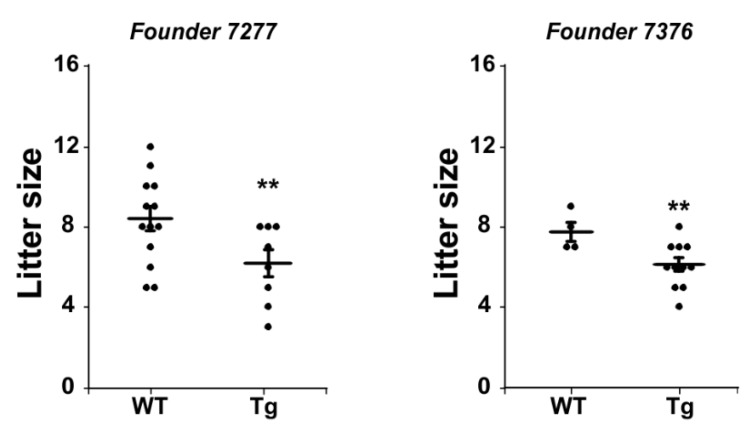
CMV-hDENND1A.V2 Tg mice display modestly lower fecundity compared to the wild-type littermates. To test for fertility outcomes, wild-type and transgenic females were bred with wild-type males. Transgenic females show lower fecundity compared to wild-type females. ** For the 7277 founder, *p* = 0.03, and for 7376, *p* = 0.02.

Histological evaluation of ovaries did not reveal notable differences in the tissue between transgenic and wild-type mice (Figure 3A). The ovaries of both transgenic and wild-type mice had luteinized follicles and relatively similar interstitial tissue after PMSG/hCG stimulation. Ovarian weights of PMSG-hCG stimulated ovaries were not different between wild-type and transgenic mice for any of the progeny from the three founder lines (Figure 3B). Although these transgenic female mice were fertile, some lines had a lower number of pups born to transgenic females bred with wild-type males in comparison to wild-type females (Figure 4). Combined, these data support the conclusion that the ovaries and adrenals of hDENND1A.V2 transgenic mice express sufficient hDENND1A.V2 to alter androgen production, and mirror the PCOS-like phenotype we previously described in cultured human theca cells with forced expression of hDENND1A.V2 [8].

**Figure 5:**
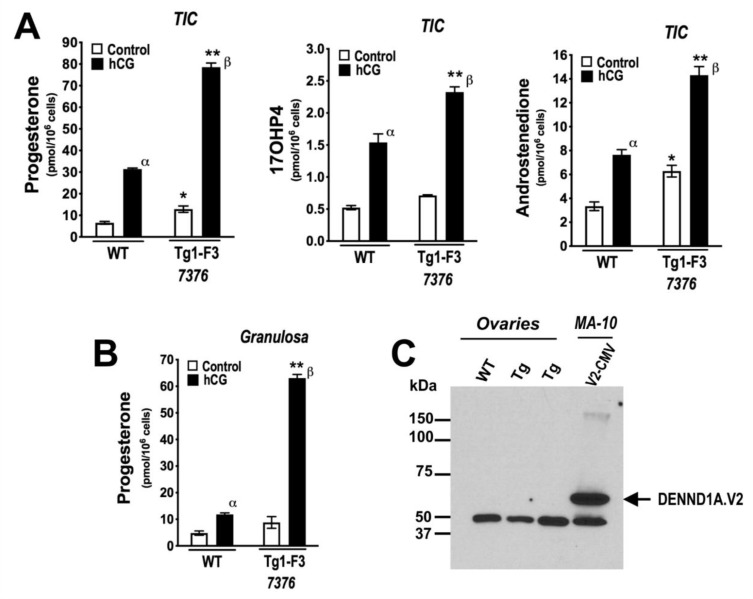
Progestin and androgen biosynthesis are increased in theca interstitial (TIC) and granulosa (GC) cells isolated from CMV-hDENND1A.V2 transgenic mice. (**A**) Examination of progesterone, 17OHP4, and androstenedione biosynthesis by cultured TIC from wild-type and F3 hDENND1A.V2 founder 7376 transgenic mice (Tg1-F3 7376), showed increases in the levels of non-stimulated (C) progesterone (*, *p* < 0.005) and androstenedione biosynthesis (*, *p* < 0.005), by CMV-hDENND1A.V2 transgenic mouse cells compared to wild-type TICs. hCG treatment (0.1 IU/mL) increased the levels of progesterone (**, *p* < 0.0001), 17OHP4 (**, *p* < 0.0001), and androstenedione (**, *p* < 0.0001) biosynthesis, as compared to wild-type TICs. hCG-treatment of TICs from both wild-type (α, *p* < 0.0001) and hDENND1A.V2 (β, *p* < 0.0001) mice similarly increased the levels of progesterone, 17OHP4, and androstenedione. (**B**) Progesterone production by cultured granulosa cells from F3 generation wild-type and Tg1-F3 7376 mice treated with and without hCG (0.1 IU/mL) for 48 h, demonstrated an increase in hCG-stimulated progesterone production (**, *p* < 0.0001). (**C**) Representative immunodetection of hDENND1A.V2 on Western blots of whole ovarian extracts from the 7277 founder line. A total of 100 μg protein was loaded in the wells containing samples from mouse ovaries and 20 μg for MA-10 cells. Expression of hDENND1A.V2 protein was not detectable in CMV-hDENND1A.V2 transgenic mice compared to extracts of MA-10 cells transfected with the pCMV-BAM-hDENND1A.V2 vector. WT, wild-type; Tg, transgenic; B, blank; V2.CMV, pCMV-BAM-hDENND1A.V2 vector; hCG, human chorionic gonadotropin; TIC, theca interstitial cells; F3, third generation.

**Figure 6:**
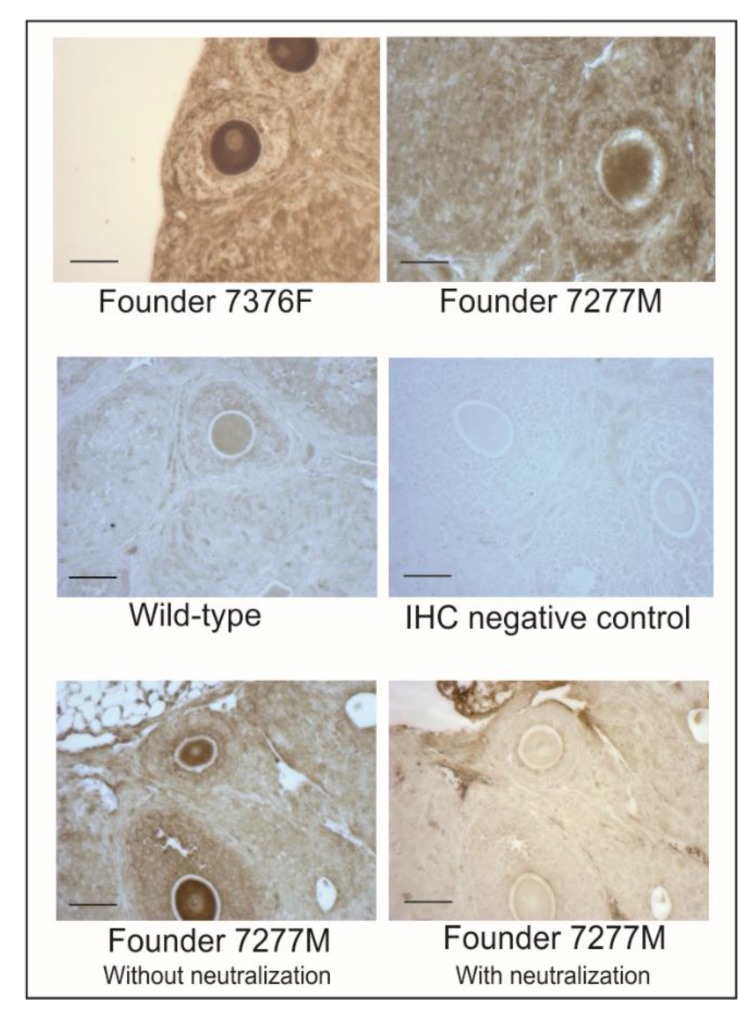
Immunohistochemical detection of hDENND1A.V2. Immunohistochemical studies were performed in ovaries from wild-type and CMV-hDENND1A.V2 transgenic mice using an anti-hDENND1A.V2 rabbit antibody. The figure shows representative images from the founder 7376F, founder 7277M, wild-type, and negative control incubated without a primary antibody. Samples were processed in parallel and all the sections were incubated in the peroxidase reaction for exactly four minutes in order to increase the detection of the signal. As expected, due to the global expression of the CMV promoter, immunoperoxidase staining is seen in all compartments in the transgenic ovaries, with a much greater intensity than in the wild-type ovary or the negative control. Validation of the specificity of the hDENND1A.V2 primary antibody was performed by neutralization of the antibody with 10 μg/mL of an hDENND1A V2-specific peptide. Bar = 50 μm.

In vitro studies to evaluate progestin and androgen production were performed in isolated theca-interstitial cell (TIC) and granulosa cell (GC) cultures from the ovaries of wild-type and CMV-hDENND1A.V2 founder 7376F line (F3 generation) mice. As shown in Figure 5A, triplicate cultures of TIC cells from CMV-hDENND1A.V2 7376 Tg mice showed a significant increase in the levels of basal (C) progesterone and androstenedione biosynthesis, compared to wild-type cells. Activation of the LH receptor with 0.1 IU/mL hCG for 48 h significantly increased the levels of progesterone, 17OHP4, and androstenedione biosynthesis in hDENND1A.V2 TICs, compared to wild-type TICs. Similarly, progesterone biosynthesis was significantly increased in cultured hDENND1A.V2 transgenic GC compared to wild-type cells (Figure 5B). Despite the functional assays suggesting a biological response to hDENND1A.V2 expression in the CMV-hDENND1A.V2 mice, we could not detect hDENND1A.V2 protein by Western blot and immunodetection (Figure 5C, Supplementary Material 6). However, the relative expression of hDENND1A.V2 mRNA in transgenic mice ovaries and TICs was >1000-fold lower than those observed in theca cells isolated and cultured from normal cycling women [8]. Thus, it is not surprising that hDENND1A.V2 protein was not visible by immunodetection on Western blots. On the other hand, we were able to detect expression of hDENND1A.V2 in the ovaries from transgenic mice by immunohistochemistry (Figure 6, Supplementary Material 7). As expected, due to the global expression of the CMV promoter, immunoperoxidase staining was present in all compartments of the transgenic ovaries, with a much greater intensity than in the wild-type ovary or the negative control and neutralized antibody control.

**Figure 7:**
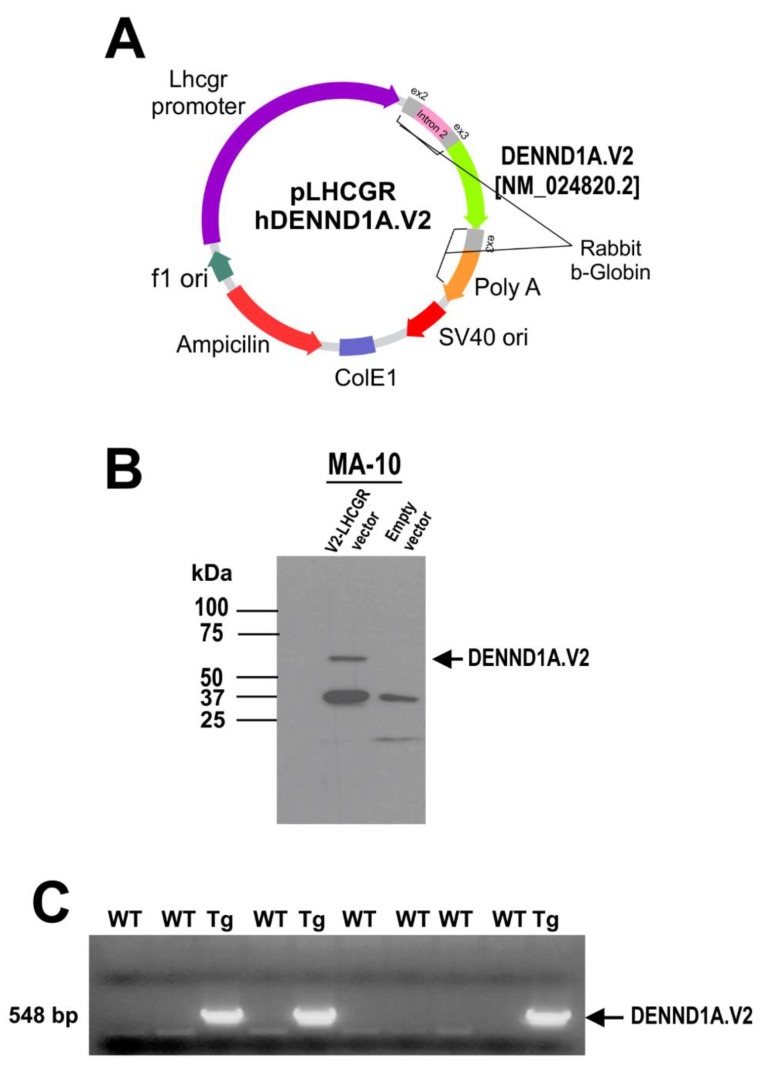
The p*Lhcgr*-hDENND1A.V2 expression construct. (**A**) Map of the p*Lhcgr*-hDENND1A.V2 construct (not drawn to scale). (**B**) Efficiency of the construct was tested by Western blot in transfected MA-10 cells using an anti-hDENND1A.V2 rabbit antibody. (**C**) Representative PCR results for the genotyping of wild-type and transgenic mice generated using the p*Lhcgr*-hDENND1A.V2 vector. WT, wild-type; Tg, transgenic; V2.*Lhcgr*, p*Lhcgr*-hDENND1A.V2 vector.

**Figure 8:**
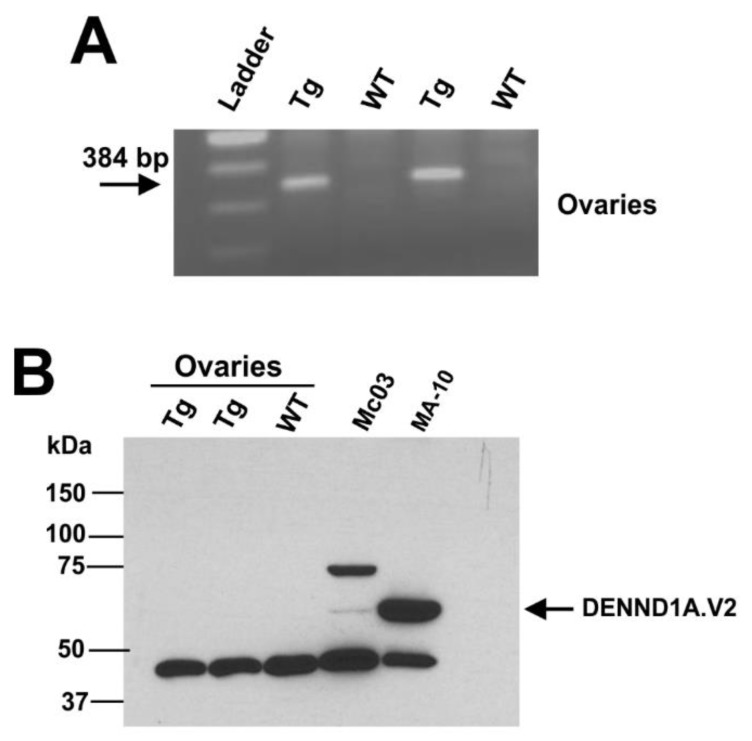
The *Lhcgr*-hDENND1A.V2 transgenic mouse profile. (**A**) Representative RT-PCR results from ovaries collected from wild-type and transgenic mice. (**B**) Representative immunodetection on Western blots for hDENND1A.V2 protein in transgenic mice. A total of 100 μg protein was loaded in the wells containing protein extracts from whole mouse ovaries, 25 μg for Mc03, and 20 μg for MA-10 cells. WT, wild-type; Tg, transgenic; Mc03 human theca cells, cultured human theca cells from a PCOS subject (positive control); MA-10, transfected cells using the p*Lhcgr*-hDENND1A.V2 construct (positive control).

hDENND1A.V2 transgenic mice using the mouse *Lhcgr* promoter to drive expression were created using a similar strategy (Figure 7A). MA-10 cells were cultured and transfected with p*Lhcgr*-hDENND1A.V2 or an empty vector, then harvested and analyzed for hDENND1A.V2 protein expression by Western blotting. A band at the expected molecular weight was present in cells transfected with the construct but absent in cells transfected with the empty construct (Figure 7B). These results confirm the ability of the p*Lhcgr*-hDENND1A.V2 vector to express the protein in a murine cell line. Mice generated with the p*Lhcgr*-hDENND1A.V2 vector were also able to express the hDENND1A.V2 transcript in the ovaries (Figure 8A). However, protein levels were again undetectable by immunodetection on Western blots (Figure 8B). The antibody detected hDENND1A.V2 in both positive controls, Mc03 cultured human theca cells from a PCOS patient, and MA-10 transfected cells using the p*Lhcgr*-hDENND1A.V2 construct. There was also cross-reactivity with a non-specific protein band at 45 kDa that served as an internal loading control. In addition, we were not able to detect *Cyp17a1* mRNA in the ovary and adrenal tissues from this transgenic line.

**Figure 9:**
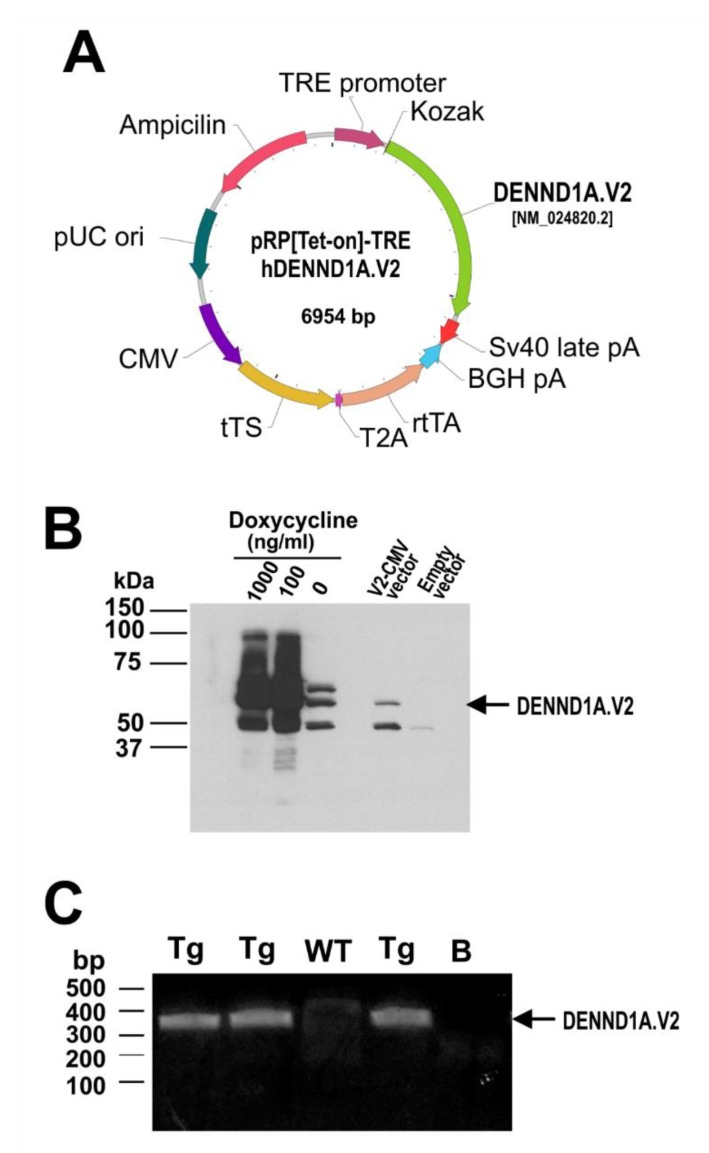
The pRP[Tet-on]-TRE-hDENND1A.V2 expression construct. (**A**) Map of the pRP[Tet-on]-TRE-hDENND1A.V2 vector. (**B**) Efficiency of the construct was tested by Western blot and immunodetection of extracts of transfected CHO cells using an anti-human DENND1A.V2 rabbit antibody. Cells transfected with the pRP[Tet-on]-TRE-hDENND1A.V2 vector were treated with 1000, 100, and 0 ng/mL of doxycycline for 48 h. The pCMV-BAM-hDENND1A.V2 vector was used as the positive control. (**C**) Representative PCR results for the genotyping of wild-type and transgenic mice generated using the pRP[Tet-on]-TRE-hDENND1A.V2 vector. WT, wild-type; Tg, transgenic; B, blank; V2.CMV, pCMV-BAM-hDENND1A.V2 vector.

**Figure 10:**
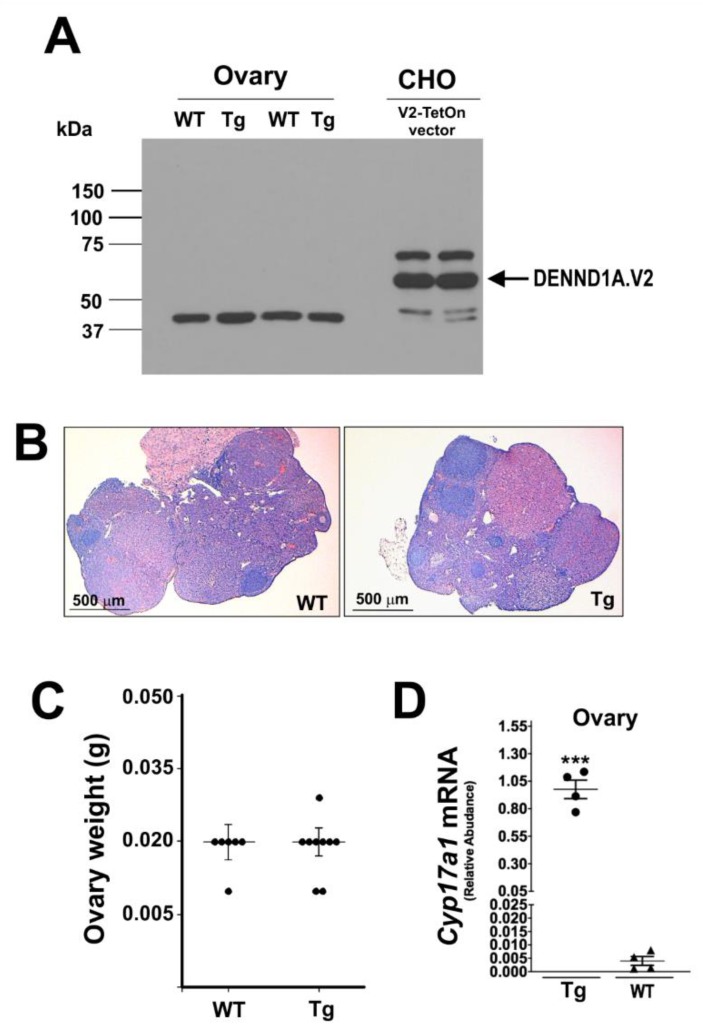
The TetOn-hDENND1A.V2 transgenic mouse profile. (**A**) Immunodetection on a Western blot for hDENND1A.V2 protein in transgenic mice. (**B**) Representative histological images from ovaries collected from wild-type and TetOn-hDENND1A.V2 transgenic mice. A total of 100 μg protein were loaded into the wells containing samples from mouse ovaries, and 10 μg for CHO cells. (**C**) Measurements of the ovary weight from wild-type and transgenic mice. No significant differences were found (*p* > 0.05). (**D**) Ovarian *Cyp17a1* mRNA levels were elevated in TetOn-hDENND1A.V2 transgenic mice compared to the WT (***, *p* = 0.0001). WT, wild-type; Tg, transgenic; V2.TetOn, pRP[Tet-on]-TRE-hDENND1A.V2 vector.

A third attempt to generate a transgenic mouse model expressing hDENND1A.V2 was made using the inducible TetOn system. The efficiency of the pRP[Tet-on]-TRE-hDENND1A.V2 construct (Figure 9A) was tested in CHO cells. Transfected CHO cells were treated with and without doxycycline for 48 h. As shown in Figure 9B, expression of hDENND1A.V2 protein using this construct was high. Transgenic animals were generated using this construct and genotyped by PCR. Amplification of DNA from transgenic mice generated a 329 bp PCR product (Figure 9C). As observed for the other transgenic models, hDENND1A.V2 protein was not detected in the ovaries from animals treated with doxycycline for 1 to 3 weeks (Figure 10A). Moreover, histological evaluation of the ovaries and ovary weight did not show differences between wild-type and transgenic mice (Figure 10B,C).

## 3. Discussion

PCOS is a common endocrine disorder characterized by androgen excess. GWAS have identified a number of loci associated with PCOS, leading to the notion that the disorder is a heritable oligogenic/polygenic condition [3,4,19,20,21]. One of the loci associated with PCOS in multiple studies of different populations includes the *DENND1A* gene. The encoded protein by this gene has a clathrin-binding domain and a DENN (Differentially Expressed in Normal and Neoplastic cells) domain, which has guanine nucleotide exchange functions. Our previous studies have revealed that theca cells from ovaries of women with PCOS produce greater amounts of androgen in vitro compared to theca cells derived from ovaries of normal cycling women [8]. The PCOS theca cells have a distinctive molecular signature that includes elevated expression of key steroidogenic enzymes, including CYP11A1 and CYP17A1 [8]. PCOS theca cells also have increased levels of a variant DENND1A transcript, termed hDENND1A.V2, an alternatively spliced mRNA that encodes a truncated protein (hDENND1A.V2), which has a unique 33 amino acid C-terminus encoded by exon 20A that distinguishes it from the longer transcript encoding DENND1A.V1 [15]. Forced expression of this variant in normal theca cells increases androgen production and expression of CYP11A1 and CYP17A1, whereas knockdown of the transcript in PCOS theca cells reduced androgen secretion and abundance of CYP11A1 and CYP17A1 mRNA [8]. Collectively, these findings suggested that increasing hDENND1A.V2 levels in theca cells is sufficient to drive androgen excess.

The present studies were conducted with the goal of creating a murine model of PCOS using transgenic technology to express hDENND1A.V2 in mouse tissues. A protein equivalent of hDENND1A.V2 does not exist in the mouse, although forced expression of hDENND1A.V2 in a mouse Leydig cell line increases steroidogenesis and *Cyp17a1* expression, demonstrating that hDENND1A.V2 is functional in the context of a mouse steroidogenic cell.

As shown in Figure S1, studies in mouse Leydig MA-10 tumor cells infected with hDENND1A.V2 adenovirus as compared to a Null (empty vector) adenovirus, demonstrated that forced expression of hDENND1A.V2 augments steroid production (17α-hydroxyprogesterone (17OHP4) and progesterone (P4), particularly in response to forskolin stimulation, which mimics the action of luteinizing hormone (LH) through activation of adenylate cyclase (Figure S1). Notably, 17OHP4 and androgen production have been previously reported to be difficult to measure in MA-10 cell media. Thus, forced hDENND1A.V2 expression in MA-10 cells boosts androgen production, to levels not previously observed [16,17]. Forced hDENND1A.V2 overexpression also increased *Cyp17a1* mRNA accumulation. These data suggest that overexpression of hDENND1A.V2 in a transgenic mouse model could phenocopy the steroidogenic abnormalities associated with human PCOS.

Here, we used three different vectors to express the hDENND1A.V2 protein in the mouse. Although, we detected expression of hDENND1A.V2 at the mRNA level, the protein expression was too low to be detected by Western blotting. The low expression level was not due to mutations in the inserted transgenes, since sequencing of genomic DNA from transgenic mice failed to identify alterations in the sequences that could explain diminished expression (Supplemental Material, Figure S2). In the future, a transgenic model might be developed using alternative technologies, like the CRISPR/Cas9 system, to introduce a hDENND1A.V2-like gene into the mouse genome. However, this assumes that the hDENND1A.V2 protein is not itself a factor preventing the creation of high expressing mice. Shi et al. reported that knockout of the mouse *Dennd1a* gene results in embryonic lethality, revealing that the DENND1A protein is essential for embryonic development and viability [22]. It is possible that the human protein interferes with critical functions of the endogenous mouse DENND1A protein, selecting against transgenic mice producing high levels of hDENND1A.V2. Such a dominant negative mechanism remains to be experimentally evaluated.

## 4. Materials and Methods

### 4.1. Adenoviral Expression of hDENND1A in Mouse MA-10 Leydig Cells

To determine whether expression of hDENND1A.V2 converts MA-10 cells to a PCOS phenotype, MA-10 were grown as previously described and were infected with 3 pfu/10^6^ cells of either null adenovirus or adenovirus expressing hDENND1A.V2. The MA-10 cells used in these studies were a generous gift from Dr. Mario Ascoli (University of Iowa). hDENND1A.V2 adenovirus (hDENND1A.V2-pADenoG) was constructed by Applied Biological Materials (Vancouver, BC, Canada), by cloning hDENND1A.V2 from pCMV6-XL4 plasmid encoding the hDENND1A.V2 into pADenoG, from Origene (Rockville, MD, USA). Control empty NULL non-expressing adenovirus (pAdenoG Null) was also obtained from Applied Biological Materials (Vancouver, BC, Canada). Recombinant adenoviruses were propagated and expanded in HEK293T cells, purified using a Virabind Adenovirus Miniprep Kit, Cell Biolabs, Inc (San Diego, CA, USA), and titered by QuickTiter Adenovirus Titer Elisa Kit, Cell Biolabs, Inc (San Diego, CA, USA). Both the DENND1A.V2 or control empty NULL non-expressing adenovirus (pAdenoG Null) were used to infect mouse Leydig MA-10 cells as we previously described [23].

### 4.2. Animals

The experiments were conducted in accordance with specific guidance and standards. The animal protocol AM10297 was approved on 1/7/19 by the Virginia Commonwealth University Institutional Animal Care and Use Committee. Before tissue collection, females were treated by intraperitoneal injection with 5 IU of PMSG (pregnant mare’s serum gonadotropin) around 1:00 p.m. to 3:00 p.m. and with 5 IU of hCG (human chorionic gonadotropin) 44 to 47 h later. Females were euthanized after 24 h of hCG treatment for tissue collection.

### 4.3. Generation of CMV-hDENND1A.V2 Transgenic Mice

To generate the CMV-hDENND1A.V2 transgene construct, the 1.7 kb human DENND1A transcript variant 2 ORF was amplified by PCR from pCMV6-XL5/DENND1A (OriGene #SC111995, OriGene Technologies, Inc. Rockville, MD, USA), and inserted in the unique *BamHI* site in the pCMV-Bam vector (a gift of Dr. Arnold Levine). The pCMV-Bam vector possesses the HCMV immediate-early promoter/enhancer followed by rabbit β-globin sequences designed to enhance transgene expression (Figure 1A). A 3.8-kb fragment containing the CMV-hDENND1A.V2 transgene was excised from the construct by digestion with *PvuI* and *EagI*, and microinjected at a concentration of 2 ng/μL into the pronucleus of fertilized C57BL/6 eggs. The injected eggs were then re-implanted into the oviducts of pseudopregnant CD-1 female mice. Potential founders were screened for the presence of the CMV-hDENND1A.V2 transgene by PCR analysis of genomic tail DNA using rabbit β-globin primers (5′-GGGGACCCTTGATTGTTCTTTC-3′) and DENND1A.V2 (5′-AGGCATGAACATGGTTAGCAGAGG-3′). Amplification of DNA from transgenic mice generated a 548 bp PCR product. In order to check for mutations along the vector we sequenced genomic DNA and cDNA from ovaries. All sequences from mouse samples were found to be identical to the sequences of the transgene constructs, indicating the absence of mutations. The sequence of ovary cDNA from a CMV-hDENND1A.V2 transgenic mouse was also determined. Once again, no mutations were detected, and the sequence exhibited precise excision of rabbit beta-globin intron (Supplementary Material 3). Three founder mice were obtained and bred to establish independent lines of mice.

### 4.4. Generation of Lhcgr-hDENND1A.V2 Transgenic Mice

The *Lhcgr*-hDENND1A.V2 transgene (Figure 6) is essentially identical to the CMV-hDENND1A.V2 transgene shown in Figure 1A, except that the HCMV promoter/enhancer was replaced with the mouse *Lhcgr* promoter. To generate the *Lhcgr*-hDENND1A.V2 transgene construct, a fragment containing 8.5 kb of the mouse *Lhcgr* promoter was obtained by partial *NotI* and *MfeI* digestion of plasmid LHR-7.4/β-GAL, and ligated to a 2.5-kb fragment obtained by digesting the CMV-hDENND1A.V2 transgene construct with *MfeI* and *EagI*. An 11.5-kb fragment containing the *Lhcgr*-hDENND1A.V2 transgene was excised from the resulting construct by digestion with *EagI,* and transgenic mice were generated and screened as described above. In order to check for mutations along the vector we sequenced genomic DNA. All sequences from the mouse samples were found to be identical to the sequences of the transgene constructs, indicating the absence of mutations (Supplementary Material 4). Only one transgenic line was developed from this construct and used for the experiments.

### 4.5. Generation of TetOn-hDENND1A.V2 Transgenic Mice

The TetOn transgene vector was generated by VectorBuilder Inc. (Santa Clara, CA, USA) by inserting the hDENND1A.V2 (NM_024820.2) sequence into a Tet-On inducible vector. Transgene animals were generated by Cyagen Biosciences Inc. (Santa Clara, CA, USA). The transgene vector was microinjected into the pronucleus of fertilized C57BL/6 eggs. The injected eggs were then re-implanted into the oviducts of pseudopregnant CD-1 female mice. Potential founders were screened for the presence of the Tet-On-hDENND1A.V2 transgene by PCR analysis of genomic tail DNA using Transgene PCR primer F1: TTTAGTGAACCGTCAGATCGC and Transgene PCR primer R1: CAACTTGGCTAACTGTGAGGCTG. Amplification of DNA from transgenic mice generated a 329-bp PCR product. In order to check for mutations along the vector we sequenced genomic DNA. All sequences from mouse samples were found to be identical to the sequences of the transgene constructs, indicating the absence of mutations (Supplementary Material 5). Only one transgenic line was developed from this construct and used for the experiments.

### 4.6. RT-PCR

RNA was isolated from the mouse tissue with TRIzol (Invitrogen, Carlsbad, CA, USA), and total RNA was reversed transcribed with RETROscript kit (Ambion, Austin, TX, USA) according to the manufacturer’s instructions. The cDNAs were used for PCR, using primer sets for CMV Tg mice 5′-AGACGCCATCCACGCTGTTTTGAC-3′ and 5′-TTGCTGTCAATGTCAGTGAGCACG-3′, which should generate a product of 401 bp; and for LHCGR mice 5′-TAGCCACCGGAGCTCACACTCAG-3′ and 5′-5′-TTGCTGTCAATGTCAGTGAGCACG-3′, which should generate a product of 384 bp. PCR settings were repeated for 40 cycles. The amplification products were resolved on 1% agarose gel stained with ethidium bromide. As a housekeeping gene 18S was used for RT-PCR. The forward primer was 5′-GGCCCTGTAATTGGAATGAGTC-3′ and the reverse 5′-CCAAGATCCAACTACGAGCTT-3′ (Supplementary Material 8).

### 4.7. Quantitative Real-Time qRT-PCR Analyses of DENND1A.V2 and Cyp17a1

Quantitation of DENND1A.V2 mRNA and *Cyp17a1* abundance was determined using the Single Step Brilliant III Ultra-Fast qRT-PCR kit (Agilent, Santa Clara, CA, USA) following the manufacturer’s instructions, using primer and probe sets for DENND1A.V2 (forward primer 5′-TCCACATGTTGTTAAGA GACCAAAG-3′; reverse primer 5′-CCGCAAAATGGGTAATGCTT-3′); Probe (5′/56-FAM/AGCCCTGAG/ZEN/CAAAACACCATTGCAA/3IA3BkFQ/-3′), as we have previously described in detail [8]; as well as the validated mouse *Cyp17a1* and tata box binding protein (tbp) primers and probe TaqMan mRNA assays, obtained from ThermoFisher (Pleasanton, CA, USA). All experiments were performed at least 3 times, in replicate tubes. The gene specific one step PCR was carried out in duplicate for each mRNA sample and for a series of dilutions in an Agilent AriaMx Real-Time PCR System (Santa Clara, CA, USA) according to manufacturer’s instructions for this instrument as previously described [8]. qPCR settings were repeated for 40 cycles. For hDENND1A.V2 assays we used 100 ng–200 ng/rxn of mouse ovarian and adrenal mRNA, as compared to 0.1 to 0.3 ng/rxn of human theca RNA from normal cycling women, to be in the middle of our control human theca hDENND1A standard curve, with a Ct between 26 and 28. Mouse tbp mRNA used for normalization and the mean expression value for each mRNA was divided by the mean tbp expression value to normalize each sample [8].

### 4.8. Western Blot and Immunodetection

Equal amounts of protein for extracts of mouse tissue (100 µg/lane) were heated to 95 °C for 10 min in sample buffer, loaded onto 7.5% SDS-PAGE gels, electrophoretically separated, and transferred to PVDF membranes (Millipore, Billerica, MA, USA) by semi-dry transference. Membranes were blocked for 1 h in 5% milk-TTBS (BIO-RAD, Hercules, CA, USA) and then incubated overnight with the previously described hDENND1A.V2 rabbit antibody [8,9], which recognizes the unique amino acid C-terminus of hDENND1A.V2. The 20 amino acid peptide ([C]-QKSITHFAAKFPTRGWTSSSH) was used to generate the antibody. Specificity of the antibody was previously validated by Kulkarni et al. [9]. After several washes in TTBS, the membranes were incubated with an anti-rabbit IgG horseradish-peroxidase labeled antibody (1:2000 dilution) for 1 h at room temperature. Protein was detected with Super Signal Chemiluminescent Substrate (Thermo Scientific, Rockford, IL, USA).

### 4.9. Cell Transfection

COS-I, CHO, and MA-10 cells were cultured in tissue culture dishes (Corning, NY, USA) for Western blot studies. COS-I and CHO were grown in DMEM, high glucose pyruvate medium (Thermo Fisher Scient ific, Waltham, MA, USA) supplemented with 10% fetal bovine serum (Thermo Fisher Scientific, Waltham, MA, USA Catalogue number: 10437028), 1% glutamine, and 1% penicillin/streptomycin (Thermo Fisher Scientific, Waltham, MA, USA). MA-10 cells were cultured on Waymouth’s media supplemented with 1.1g/l NaHCO_3_, 20mM HEPES, 50 mg/mL gentamicin, and 15% horse serum. At 60–70% confluency cells were transfected with pCMV-BAM-hDENND1A.V2, pLHCGR-hDENND1A.V2, pRP[Tet-on]-TRE-hDENND1A.V2, or an empty vector control (pCMV-BAM) using Continuum (Gemini Bio-products, West Sacramento, CA, USA). Cells were harvested 48 h after transfection.

### 4.10. Immunofluorescence Detection of hDENND1A.V2

CHO cells transfected with pCMV-BAM-hDENND1A.V2 vector were fixed with 4% formalin for 1 h, washed with PBS three times and incubated for 1 h at room temperature with blocking solution containing 10% goat serum (Vector Laboratories, Inc., Burlingame, CA, USA), 3% BSA (Sigma-Aldrich, St. Louis, MO, USA), and 0.2% Triton X-100 (Sigma-Aldrich, St. Louis, MO, USA). After blocking, cells were incubated overnight at 4 °C with anti-hDENND1A.V2 rabbit polyclonal antibody (dilution 1:100) [8,9]. After washing, the cells were incubated with anti-rabbit Cy3-labeled secondary antibody (Jackson ImmunoResearch Laboratory Inc, Grove, PA, USA) for 1 h at room temperature. For mounting VectaMount with DAPI (Vectorsheild, Vector Laboratories Inc., Burlingame, CA, USA) was used. Images were captured by a Zeiss LSM 700 confocal laser-scanning microscope.

### 4.11. Immunohistochemical Detection of hDENND1A.V2

Immunohistochemical (IHC) analysis for hDENND1A.V2 was performed on 5 μm-thick sections of formalin-fixed, paraffin-embedded ovarian tissue collected from PMSG/hCG-stimulated wild-type and transgenic mice.

Slides containing the sectioned ovarian tissue were deparaffinized in 100% xylene and then rehydrated through decreasing the concentration of the ethanol. The slides were then treated with 3% H_2_O_2_ in methanol for 30 min to remove endogenous peroxidase. Antigen retrieval was performed in citrate buffer at 95 °C for 20 min. After washing several times in PBS, slides were incubated with blocking solution containing 10% goat serum and 0.2% triton X-100 in 1X PBS for 1 h at room temperature. A primary anti-hDENND1A.V2 rabbit polyclonal antibody was used for overnight incubation at 4 °C, 1:200 dilution. Then, slides were incubated with biotinylated secondary anti-rabbit antibody (Vector laboratories, Burlingame, CA, USA) for 1 h at room temperature. Following the incubation, the slides were washed with PBS and incubated with Vectastain Avidin-Biotin-Complex (ABC) reagent (Vector Laboratories Inc., Burlingame, CA, USA) for 30 min at room temperature. Following several washes with PBS, the staining was developed with ImmPACT Diaminobenzidine (DAB) for 2 min and rinsed with tap water to stop the reaction. Wild-type and transgenic samples were processed in parallel to ensure the same conditions for these samples. Lastly, the stained slides were dehydrated using an increasing concentration of ethanol and xylene and were mounted with Vectastain (Vector Laboratories Inc., Burlingame, CA, USA) mounting media. The primary antibody was neutralized by preincubation with the hDENND1A V2 specific peptide ([C]-QKSITHFAAKFPTRGWTSSSH; ChinaPeptides, Shanghai, China) for 30 min at room temperature. The peptide concentration used was 10 μg/mL.

### 4.12. Theca-Interstitial Cell (TIC) and Granulosa Cell (GC) Isolation

TIC and GC were isolated from F3 wild-type and CMV-hDENND1A.V2 Tg mouse ovaries and cultured as described by Tian et al. [24]. Briefly, ovaries were aseptically removed from wild-type and transgenic mice and placed in ice-cold Lebovitz’s-15 medium (Invitrogen/Thermo Fisher Scientific, Waltham, MA, USA) supplemented with 1mg/mL bovine serum albumin (Sigma-Aldrich, St. Louis, MO, USA) and 1% penicillin/streptomycin (Thermo Fisher Scientific, Waltham, MA, USA). Ovaries were cleaned of the surrounding marginal tissue and washes several times with the same media. GC were collected by puncturing ovary follicles with a 25G needle and then cultured for 48 h at 37 °C, 5% CO_2_, and 95% humidity in McCoy’s 5a medium supplemented with 5% FBS and 1% penicillin/streptomycin (Thermo Fisher Scientific, Waltham, MA, USA). The remaining ovary tissue was washed with media and digested for 60 min at 37 °C in collagenase-DNAse solution containing 4 mg/mL collagenase IV (Sigma-Aldrich, St. Louis, MO, USA), 10 μg/mL DNAse (Sigma-Aldrich, St. Louis, MO, USA), and 10 mg/mL BSA (Sigma-Aldrich, St. Louis, MO, USA) in M199 medium (Invitrogen/ Thermo Fisher Scientific, Waltham, MA, USA) to isolate TIC. After digestion, cells were centrifuged at 1000 rpm for 5 min and washed several times in McCoy’s 5a medium (Sigma-Aldrich, St. Louis, MO, USA). Finally, TICs were resuspended and cultured for 48 h at 37 °C, 5% CO_2_, and 95% humidity in McCoy’s 5a medium supplemented with 5% FBS and 1% penicillin/streptomycin (Thermo Fisher Scientific, Waltham, MA, USA).

### 4.13. Quantitation of Progesterone (P4), 17α-hydroxyprogesterone (17OHP4), and Androstenedione (adione) by ELISA

ELISAs for P4, 17OHP4, and adione were performed without organic solvent extraction using kits from DRG International, Inc. (Springfield, NJ, USA) as described by the manufacturer’s protocol. ELISA for P4 was performed on sera collected from WT and CMV-hDENND1A.V2 Tg mice. In addition, P4, 17OHP4, and adione ELISAs were performed with cell culture media collected from ovarian theca interstitial and granulosa cells from F3 WT and CMV-hDENND1A.V2 Tg mice, treated with and without 20 μM forskolin for 48 h, and normalized by cell protein. The P4 antibody displayed 100% cross reactivity with P4; 17OHP4, 0.3%; corticosterone, 0.2%; pregnenolone 0.1%; <0.01% for DHEA, DHEAS, estriol, estradiol-17β, DHT, cortisol, testosterone (T), and adione. The 17OHP4 ELISA antibody displayed 100% cross-reactivity with 17OHP4; 11-desoxycortisol, 1.4%; P4, 1.2%; deoxycortisol, 0.05%; corticosterone, <0.05%; and <0.01% for DHEA, DHEAS, estriol, estradiol-17β, DHT, cortisol, aldosterone, and adione. The adione antibody displayed 100% cross-reactivity to adione; progesterone, 0.01%; and < 0.01% to 17OHP4; 17OHP5, DHEA, androsterone, cortisol, T, dihydrotestosterone, estriol.

### 4.14. Statistical Methods

Statistical analysis was performed with Prism 8.3 by GraphPad Software (San Diego, CA, USA). A t-test was used for comparison of two groups of data, and a one-way ANOVA with Bonferroni corrections when multiple groups of data were compared. Data were presented as means ± standard errors. Normality of data was assessed by the D’Agostino and Pearson omnibus normality test. Samples were considered significantly different when the *p*-value was < 0.05. InfoStat software [25] was also used for drawing graphs and statistical analyses shown in Figure 3B, Figure 4, and Figure 10C.

## Supplementary Materials

Supplementary materials can be found at https://www.mdpi.com/1422-0067/21/7/2545/s1.

## Abbreviations

PCOS	polycystic ovary syndrome
GWAS	Genome Wide Association Study
DENND1A	DENN (Differentially Expressed in Neoplastic vs. Normal cells) Domain Containing 1A
hDENND1A.V2	hDENND1A variant 2 (a truncated splice variant of the DENND1A gene)
*Lhcgr*	Luteinizing Hormone/Choriogonadotropin Receptor
Cyp17	mouse cytochrome P450 17-hydroxylase
P4	progesterone
17OHP4	17-hydroxyprogesterone
