# Supplementary figures and images for: Human DENND1A.V2 Drives Cyp17a1 Expression and Androgen Production in Mouse Ovaries and Adrenals
Source: Int J Mol Sci. 2020 Apr 6;21(7):2545. doi: 10.3390/ijms21072545 (PMC7177906; doi:10.3390/ijms21072545)

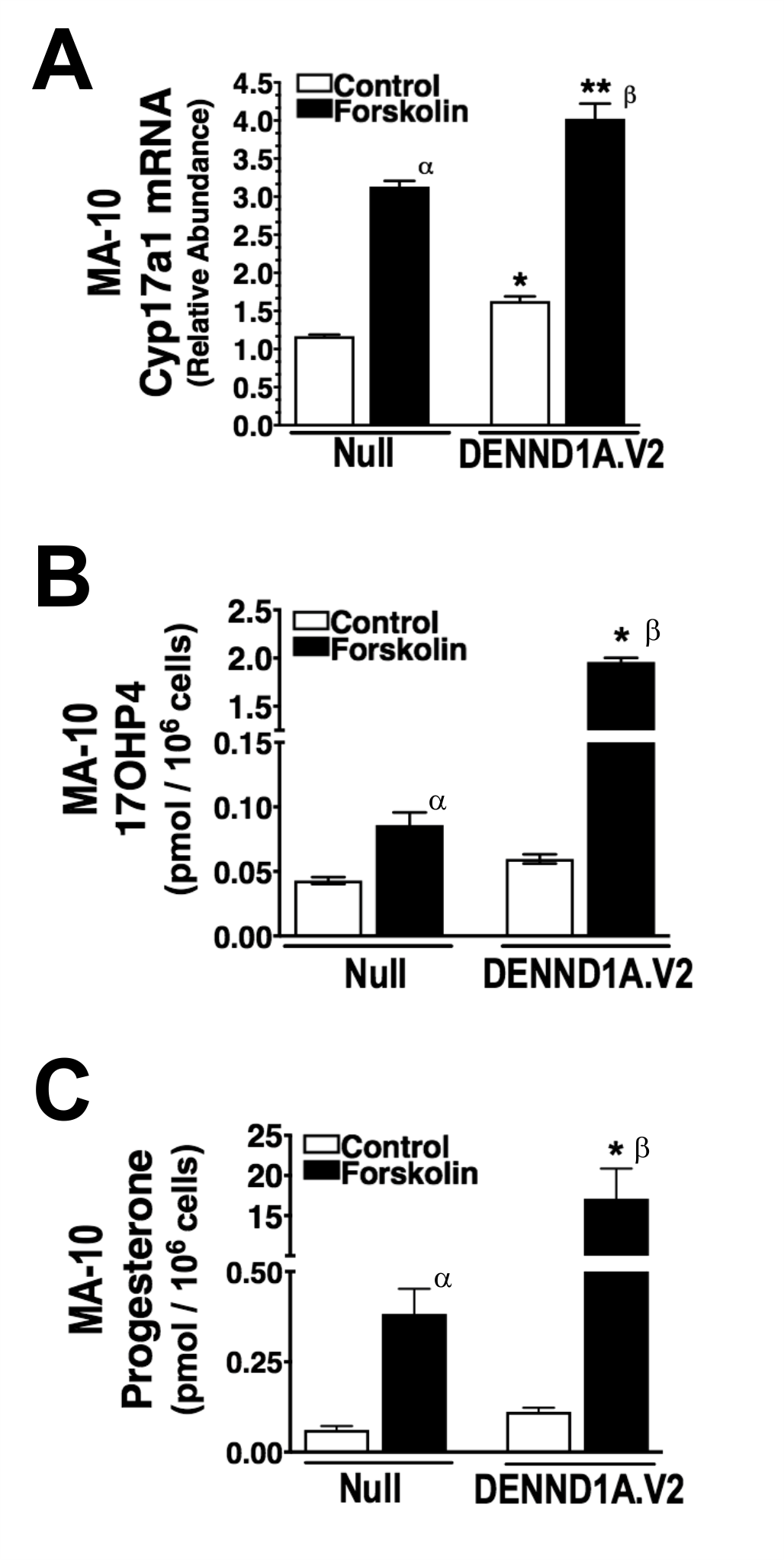

Supplement: Supplementary file 1 [file ijms-21-02545-s001.zip › Supplementary material/Fig. S1.tif]

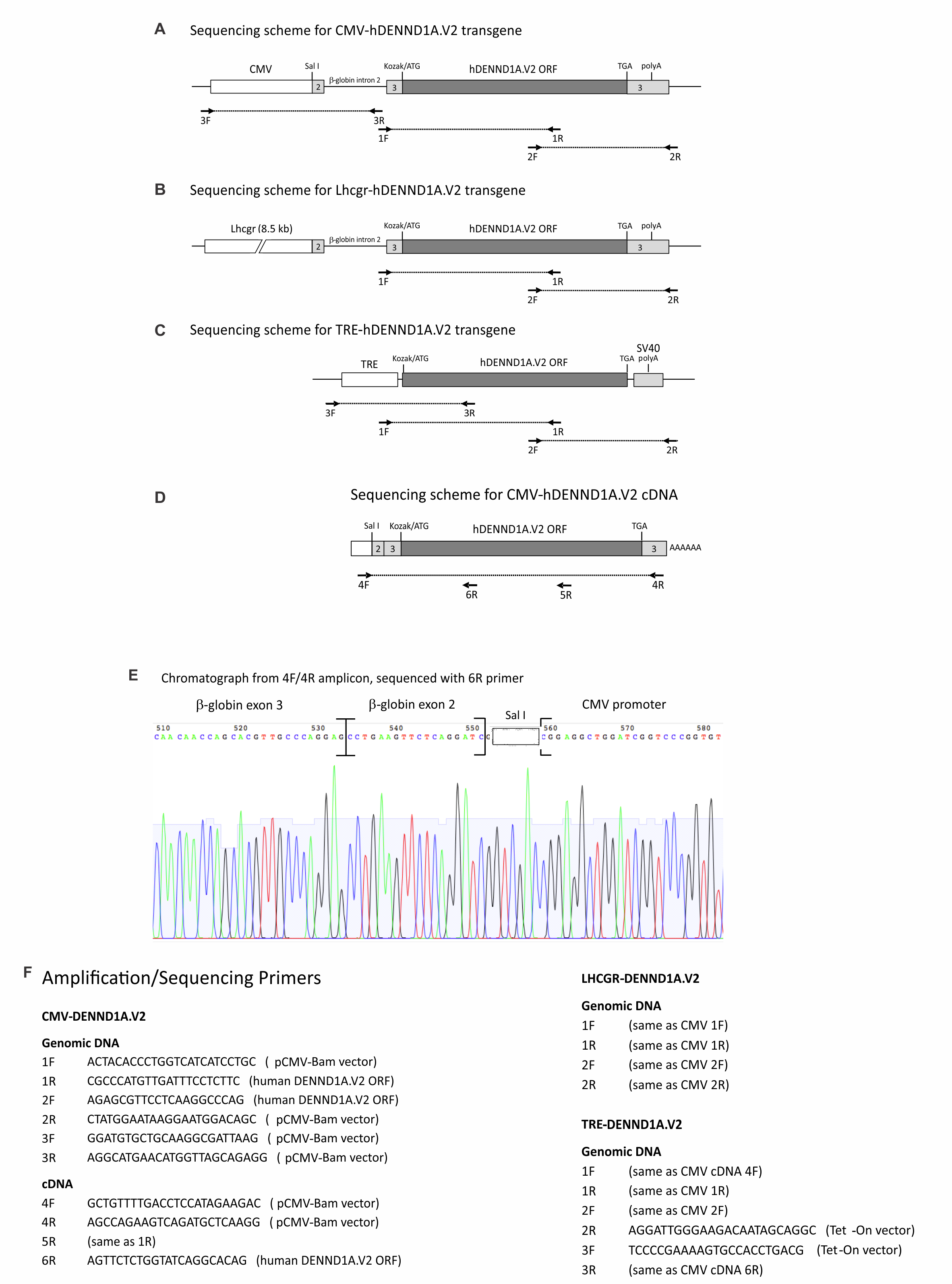

Supplement: Supplementary file 1 [file ijms-21-02545-s001.zip › Supplementary material/Figure S2.tif]

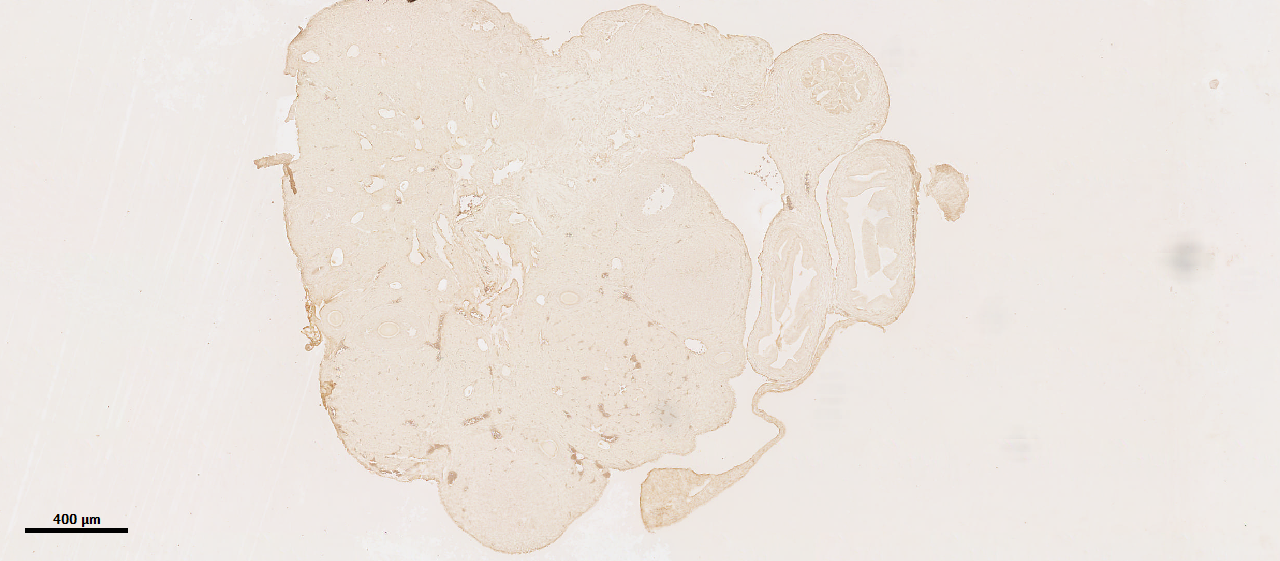

Supplement: Supplementary file 1 [file ijms-21-02545-s001.zip › Supplementary material/Supplementary material 7/Supplementary material 7. Founder 7277 with neutralization.tif]

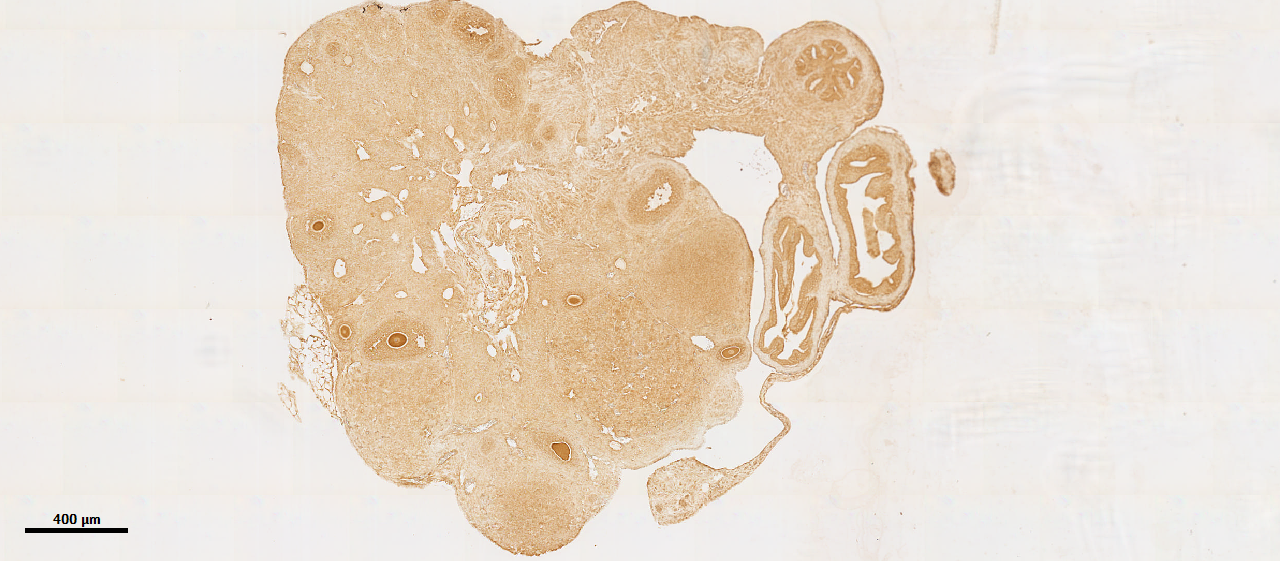

Supplement: Supplementary file 1 [file ijms-21-02545-s001.zip › Supplementary material/Supplementary material 7/Supplementary material 7. Founder 7277 without neutralization.tif]

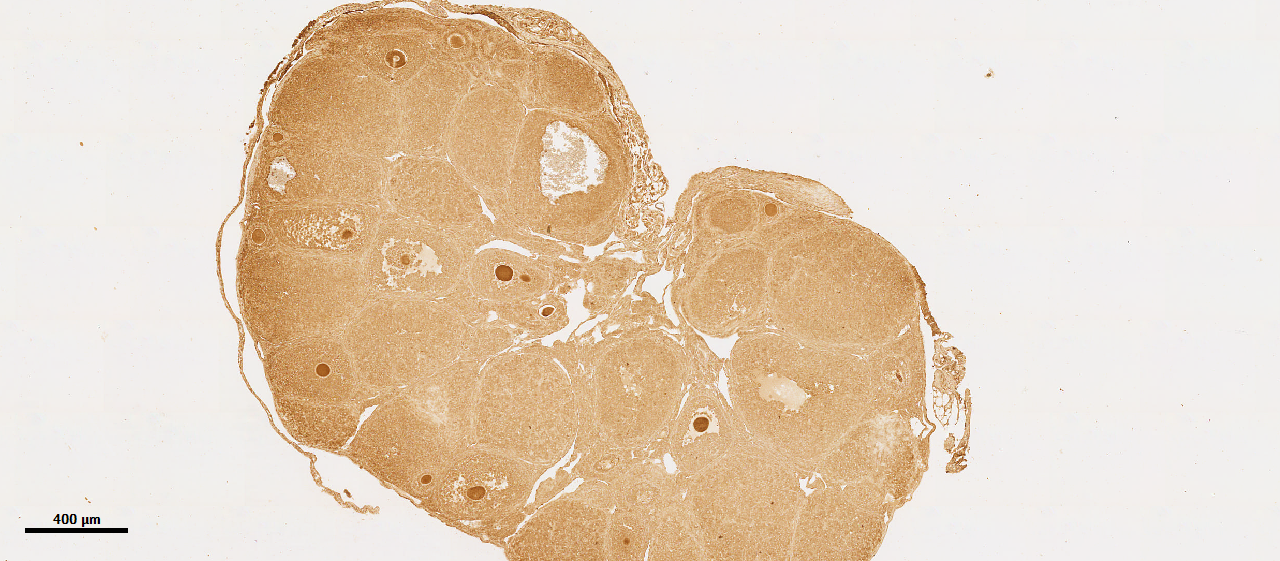

Supplement: Supplementary file 1 [file ijms-21-02545-s001.zip › Supplementary material/Supplementary material 7/Supplementary material 7. Founder 7277.tif]

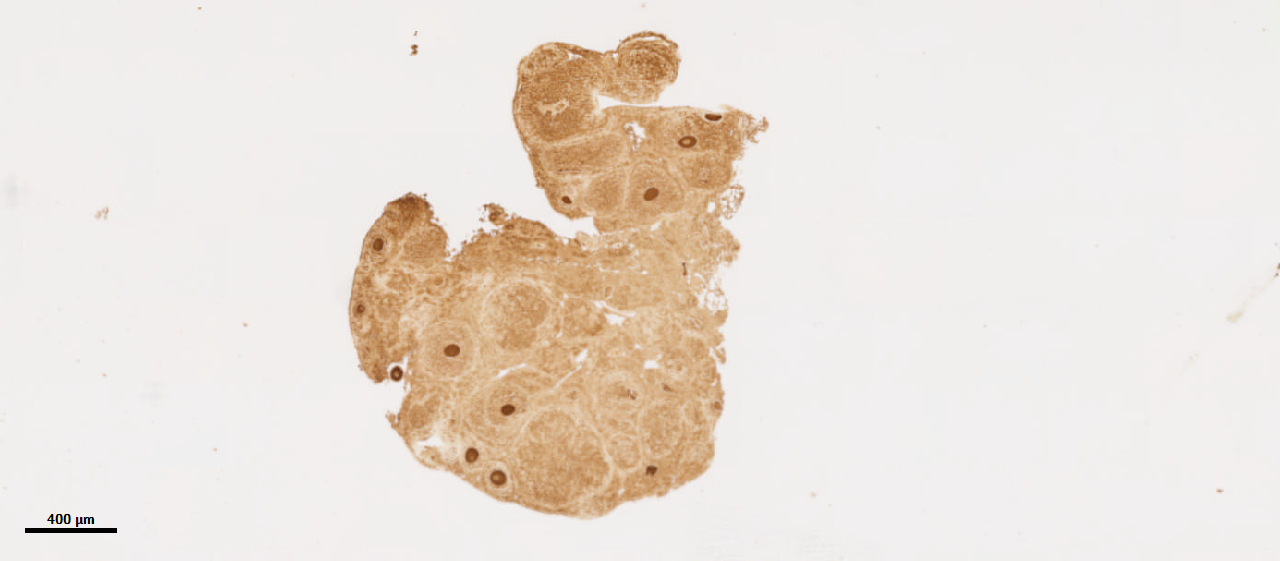

Supplement: Supplementary file 1 [file ijms-21-02545-s001.zip › Supplementary material/Supplementary material 7/Supplementary material 7. Founder 7376.tif]

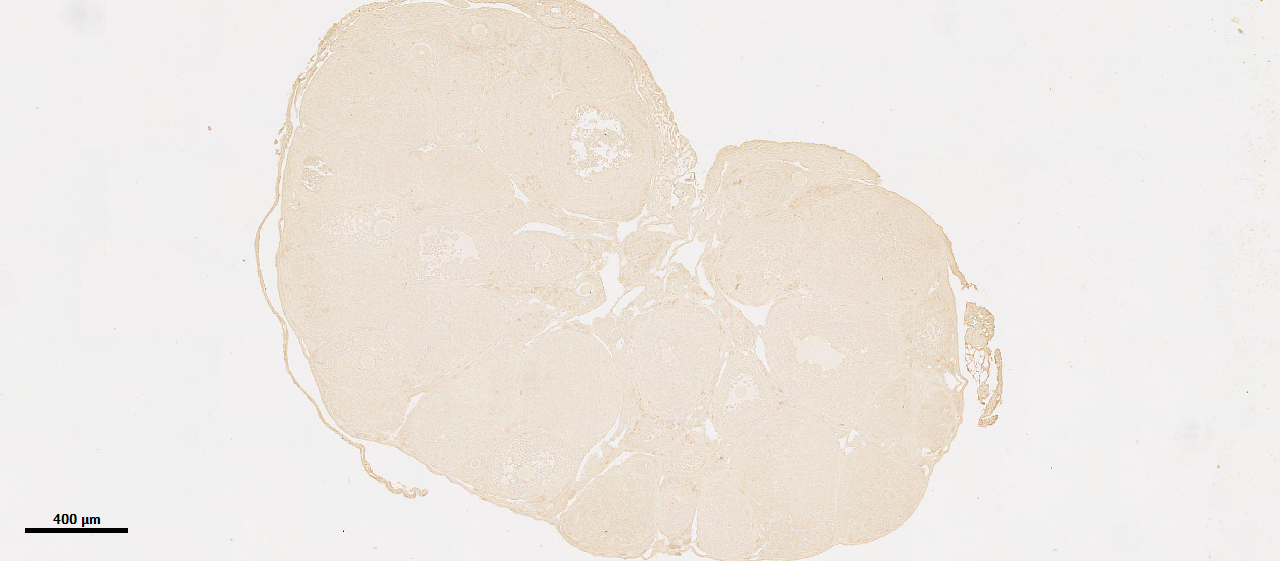

Supplement: Supplementary file 1 [file ijms-21-02545-s001.zip › Supplementary material/Supplementary material 7/Supplementary material 7. Negative control.tif]

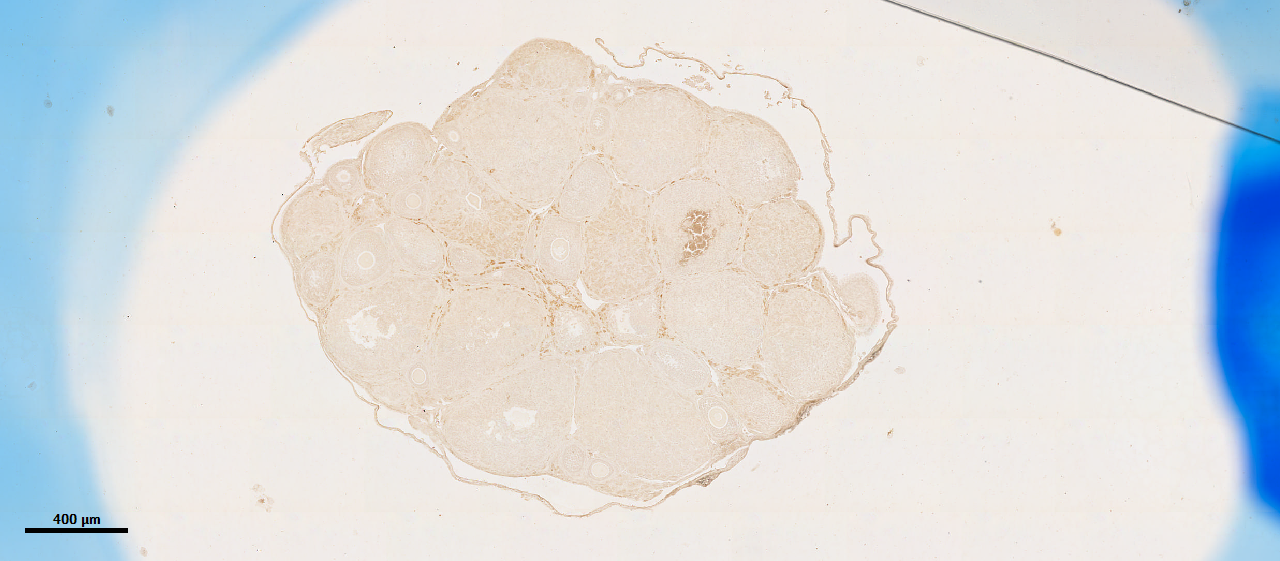

Supplement: Supplementary file 1 [file ijms-21-02545-s001.zip › Supplementary material/Supplementary material 7/Supplementary material 7. Wild-type.tif]
